# Effect of Zinc Supplementation on Renal Anemia in 5/6-Nephrectomized Rats and a Comparison with Treatment with Recombinant Human Erythropoietin

**DOI:** 10.3390/ijms20204985

**Published:** 2019-10-09

**Authors:** Hui-Lin Feng, Yen-Hua Chen, Sen-Shyong Jeng

**Affiliations:** 1 Department of Food Science, College of Life Sciences, National Taiwan Ocean University, Keelung 20224, Taiwan; fhl1430@gmail.com; 2 Department of Basic Medicine, The Center of Translational Medicine, Xiamen Medical College, Xiamen 361023, China

**Keywords:** chronic kidney disease, EPO production, 5/6-nephrectomized rats, renal anemia, zinc supplementation

## Abstract

Anemia is a severe complication in patients with chronic kidney disease (CKD). Treatment with exogenous erythropoietin (EPO) can correct anemia in many with CKD. We produced 5/6-nephrectomized rats that became uremic and anemic at 25 days post surgery. Injection of the anemic 5/6-nephrectomized rats with 2.8 mg zinc/kg body weight raised their red blood cell (RBC) levels from approximately 85% of the control to 95% in one day and continued for 4 days. We compared the effect of ZnSO_4_ and recombinant human erythropoietin (rHuEPO) injections on relieving anemia in 5/6-nephrectomized rats. After three consecutive injections, both the ZnSO_4_ and rHuEPO groups had significantly higher RBC levels (98 ± 6% and 102 ± 6% of the control) than the saline group (90 ± 3% of the control). In vivo, zinc relieved anemia in 5/6-nephrectomized rats similar to rHuEPO. In vitro, we cultured rat bone marrow cells supplemented with ZnCl_2_, rHuEPO, or saline. In a 4-day suspension culture, we found that zinc induced erythropoiesis similar to rHuEPO. When rat bone marrow cells were supplement-cultured with zinc, we found that zinc stimulated the production of EPO in the culture medium and that the level of EPO produced was dependent on the concentration of zinc supplemented. The production of EPO via zinc supplementation was involved in the process of erythropoiesis.

## 1. Introduction

Anemia is a severe complication in patients with chronic kidney disease (CKD). Erythropoietin (EPO) deficiency is the major cause [1] because the kidneys of CKD patients are not able to produce sufficient EPO to stimulate RBC formation (erythropoiesis). Treatment with exogenous EPO, e.g., rHuEPO, can correct anemia in many with CKD [2].

Zinc in kidney disease has been reviewed by several investigators [3,4,5,6,7]. Abnormalities in zinc metabolism are well documented in CKD patients [3,4]. A systematic review of hemodialysis patients showed that the zinc level was lower in hemodialysis patients, compared with controls [5], and zinc deficiency was highly prevalent [7]. Zinc deficiency not only showed an influence on inflammatory and immunological processes, but also interfered with metabolism and other systems [6,7]. Lower plasma zinc levels have been found to be related to renal disease. Furthermore, zinc supplementation to CKD patients was considered beneficial, resulting in higher plasma zinc levels and benefiting the nutritional status of maintenance hemodialysis patients [7,8,9,10]. Zinc supplementation was found to increase hemoglobin and hematocrit levels in hemodialysis patients [11]. Adjuvant zinc therapy has been used for zinc-deficient anemic patients, and zinc was found to be an economic choice to reduce the use of rHuEPO in CKD patients on hemodialysis [12,13]. Although micronutrients, including zinc, are known to be relevant to blood cell production, some of the mechanisms remain unknown [14].

In our previous study [15], we induced anemia in rats using phenylhydrazine (PHZ) and injected either saline or ZnSO_4_ solution. We found that when the concentration of ZnSO_4_ was higher than 2.8 mg zinc/kg body weight, the RBC level of the anemic rats increased from 60 ± 7% to 88 ± 10% that of the normal rats in two days. Rat bone marrow cells cultured with 0.3 mM ZnCl_2_ had a 1.6-fold proliferation of nascent immature reticulocytes (new RBCs) in one day. Zinc supplementation could relieve anemia in PHZ-induced rats in vivo and in vitro. Since zinc is able to relieve anemia in PHZ-induced rats, we speculated that the ability of zinc to reduce the use of rHuEPO in CKD patients on hemodialysis mainly occurred because zinc supplementation induced erythropoiesis in these patients. However, there is no animal experiment to verify that zinc supplementation induces erythropoiesis in renal anemic rats, and no explanation has been suggested as to why zinc supplementation could relieve renal anemia.

Five-sixth-nephrectomized rats mimic the progression of renal failure after the loss of renal mass in humans and has been a widely used experimental model of CKD [16,17,18]. The effect of EPO on 5/6-nephrectomized rats has been reported previously [19,20]. In the present report, we produced 5/6-nephrectomized rats and injected them with ZnSO_4_ or saline to determine their effect on erythropoiesis in the rats. We also compared the effect of treatment with rHuEPO and found that zinc supplementation had the same effects as rHuEPO in increasing RBC counts in the 5/6-nephrectomized rats. In vitro, we cultured rat bone marrow cells with ZnCl_2_, rHuEPO, or saline and found that zinc induced erythropoiesis similar to rHuEPO. This zinc-induced erythropoiesis in the rats might come from the production of EPO from the bone marrow cells.

## 2. Results

### 2.1. Effect of ZnSO_4_ Injection on the RBC Level of 5/6-Nephrectomized Rats

**Figure 1:**
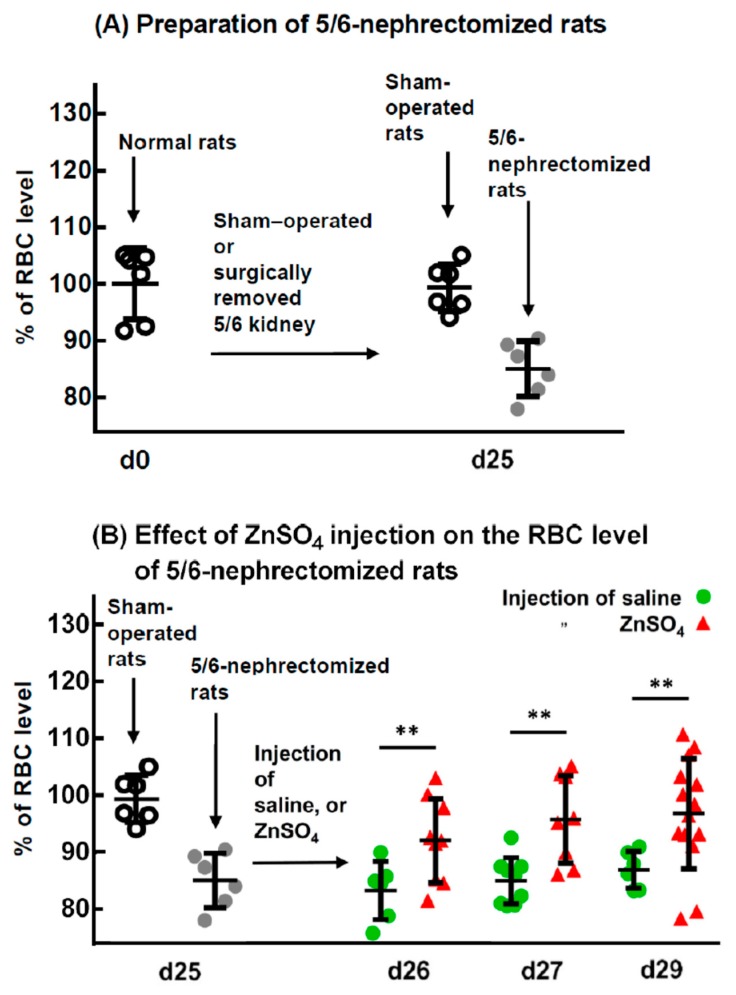
Preparation of 5/6-nephrectomized rats and the effect of ZnSO_4_ injection on their red blood cell (RBC) levels. (**A**) Normal rats underwent a sham-operation or surgical removal of 5/6 of the kidney. After 25 days, rat blood was drawn, and RBC levels and other hematological indices of the rats were measured. (**B**) Effect of ZnSO_4_ injection on the RBC level of 5/6-nephrectomized rats. The 5/6-nephrectomized rats (25 days post surgery) were injected with either saline or 2.8 mg Zn/kg body weight of ZnSO_4_ solution. One, 2, or 4 days after injection, RBC levels and other hematological indices were measured. ** Significant difference between ZnSO_4_-injected and saline-injected rats (*p* < 0.01, *n* = 6). This figure is representative of three independent experiments.

Figure 1A indicates that 25 days after a sham operation on the kidneys of the rats, the RBC levels of the rats were 99 ± 4% of those before operation (at day 0, 100%), ((7.57 ± 0.32) × 10^6^ vs. (7.62 ± 0.47) × 10^6^ cells/mm^3^). These results indicate that the sham operation of the kidney had no effect on the RBC level of the rats. However, the RBC levels of the 5/6-nephrectomized rats were significantly lower than those of the controls (sham-operated rats), at only 85 ± 4% of the control ((6.48 ± 0.37) × 10^6^ vs. (7.57 ± 0.32) × 10^6^ cells/mm^3^). To determine the effect of zinc on the RBC counts of rats that had already become uremic and anemic, 5/6-nephrectomized rats (25 days post surgery) were injected with ZnSO_4_ or saline. After 1, 2, and 4 days, the RBC counts of the ZnSO_4_-injected groups were all significantly higher than those injected with saline, i.e., 92 ± 7 vs. 83 ± 5%, 96 ± 8 vs. 85 ± 4%, and 97 ± 10 vs. 87 ± 3%, respectively. Supplementation with an adequate amount of zinc to the 5/6-nephrectomized rats relieved their anemia in 1 day, and from 2 days until 4 days, the RBC levels returned to over 95% of those of the control.

### 2.2. New Methylene Blue Staining of the Blood Cells of 5/6-Nephrectomized Rats Injected with Either Saline or ZnSO_4_

**Figure 2:**
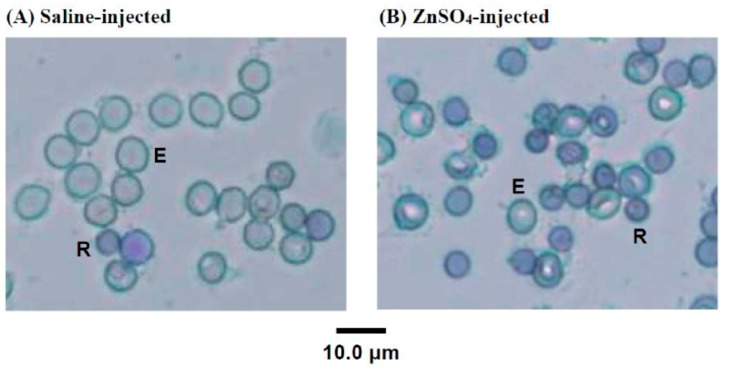
Microscopic observation of new methylene blue-stained blood cells of (**A**) saline-injected and (**B**) ZnSO_4_-injected 5/6-nephrectomized rats (27 days post surgery). In (A), most of the blood cells were mature erythrocytes (E) (median cell diameter 7.4 μm), and their cytoplasm was clear. Several smaller cells (R) were also present. In (B), the ZnSO_4_-injected 5/6-nephrectomized rats had less mature erythrocytes (E) but had more smaller cells (R) (median cell size 5.1 μm), and these cells (R) had curved linear structures or dark blue dots in the cytoplasm with distinctive staining of the reticulocytes.

To determine the change in blood cell composition in the 5/6-nephrectomized rats after the injection of ZnSO_4_, the blood cells of the above experiment (Figure 1B, d27) were stained with new methylene blue, as shown in Figure 2. Newly proliferated immature RBC cells showed reticular, mesh-like structures when observed using microscopy with new methylene blue staining, which are known as reticulocytes [21]. Figure 2 shows that many immature reticulocytes proliferated after the anemic 5/6-nephrectomized rats were injected with ZnSO_4_. The reticulocyte percentage in the saline-injected 5/6-nephrectomized rats’ blood was calculated to be 7%, while that of the ZnSO_4_-injected one was 15%.

### 2.3. Effect of ZnSO_4_ Injection on the EPO Level in the 5/6-Nephrectomized Rats

**Figure 3:**
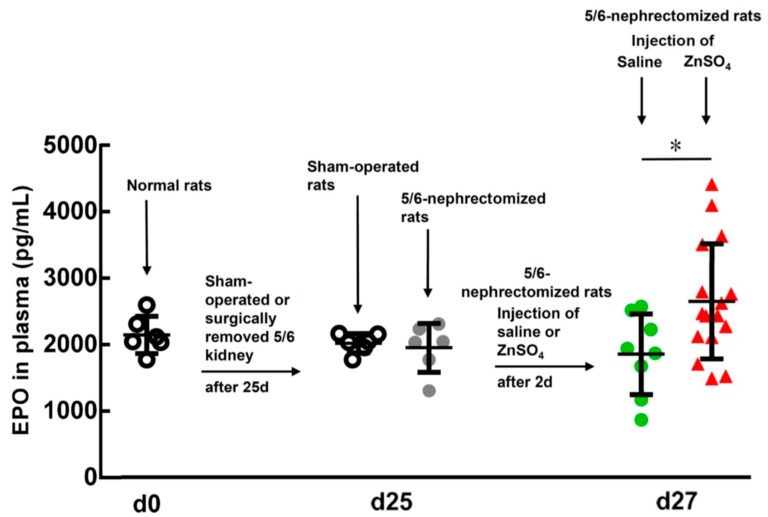
EPO levels in the plasma of normal and saline- or ZnSO_4_-injected 5/6-nephrectomized rats (27 days post surgery). EPO levels in the plasma of the rats before and after the experiment were measured using an enzyme-linked immunosorbent assay (ELISA) kit. * Significant difference (*p* = 0.014) between saline (*n* = 8)- and ZnSO_4_ (*n* = 17)-injected 5/6-nephrectomized rats.

In our experiment, the normal rats had plasma EPO levels of 2353 ± 242 pg/mL (*n* = 6) at day 0 (Figure 3). The sham-operated and 5/6-nephrectomized rats (25 days after surgery) had 1959 ± 157 (*n* = 6) and 1896 ± 722 (*n* = 6) pg/mL EPO levels, respectively (*p* = 0.327). However, 2 days after the 5/6-nephrectomized rats were injected with ZnSO_4_, their EPO levels were significantly higher than those injected with saline, 2644 ± 944 (*n* = 8) versus 1574 ± 862 (*n* = 17) pg/mL (*p* = 0.014). A 1.7-fold increase in the EPO level was observed.

### 2.4. In Vivo Comparison of the Injection of Saline, ZnSO_4_, or rHuEPO on the RBC Levels of 5/6-Nephrectomized and Normal Rats

**Figure 4:**
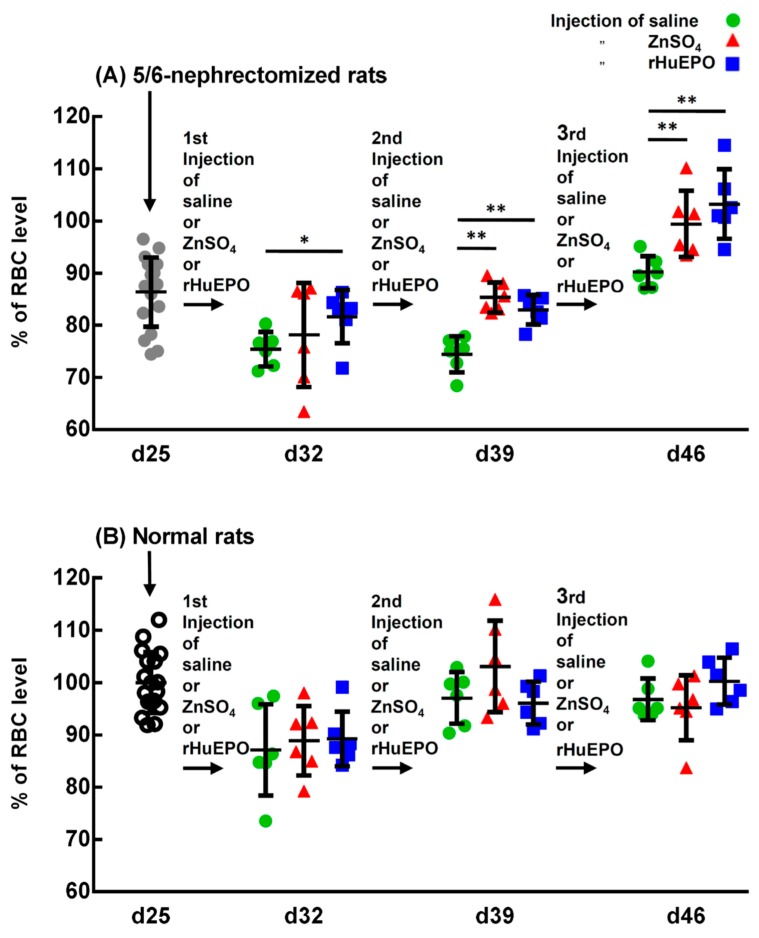
Comparison between the injection of saline, ZnSO_4_, or rHuEPO on the RBC level of 5/6-nephrectomized and normal rats. (**A**) Injection of saline, ZnSO_4_, or rHuEPO on the RBC levels of 5/6-nephrectomized rats. Rat blood was drawn from the 5/6-nephrectomized rats (25 days post surgery); then, the rats were injected with saline, ZnSO_4_, or rHuEPO. Every 7 days, blood was drawn, and saline, ZnSO_4_, or rHuEPO was injected. The collected blood was used to measure RBC levels and other hematological indices. * and **: Significant difference between ZnSO_4_- or rHuEPO- and saline-injected rats (* *p* < 0.05; ** *p* < 0.01; *n* = 6). (**B**) Injection of saline, ZnSO_4_, or rHuEPO on the RBC level of normal rats. Normal rats (same age and lot as the 5/6-nephrectomized rats) were subjected to the same procedure described above. This figure is representative of three independent experiments.

The effect of the injection of saline, ZnSO_4_, or EPO on the RBC levels of the 5/6-nephrectomized rats is shown in Figure 4A. Other hematological indices are shown in Table S1. After the second and third injections, both the ZnSO_4_ and rHuEPO injection groups had significantly higher RBC levels than those injected with saline (Figure 4A, day 39 and day 46). At day 46, the 5/6-nephrectomized rats injected with saline had RBC percentages of only 90 ± 3% compared with the control rats; however, those injected with ZnSO_4_ and rHuEPO restored their RBC percentages to 98 ± 6 and 102 ± 6%, respectively. These experiments show that, in vivo, zinc induced erythropoiesis in 5/6-nephrectomized rats similarly to rHuEPO.

To determine the effect of the injection of ZnSO_4_ or rHuEPO on the hematological indices of the normal rats, normal rats of the same age and lot to that of the 5/6-nephrectomized rats were studied in a similar way. Figure 4B indicates that in normal rats, there were no significant differences in RBC levels between the group injected with saline and the group injected with ZnSO_4_ or rHEPO. Other hematological indices that had similar results are shown in Table S2.

### 2.5. Effect of ZnSO_4_ or rHuEPO Injection on Blood Urea Nitrogen and Creatinine of Rats

**Figure 5:**
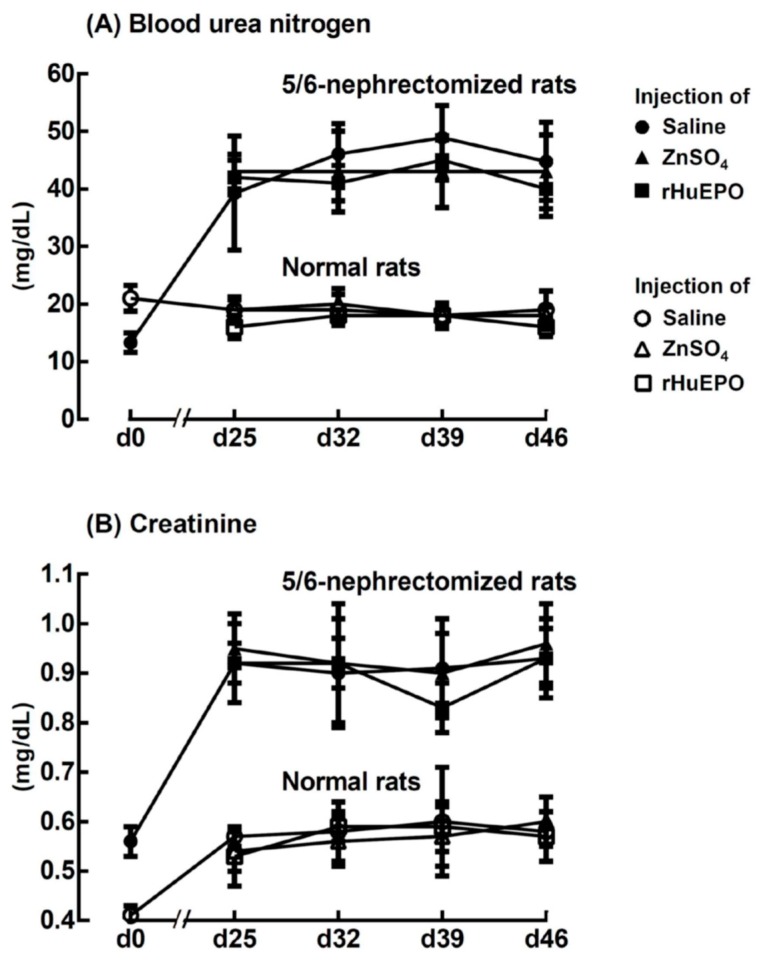
Effect of injection of saline, ZnSO_4_, or rHuEPO on (**A**) blood urea nitrogen and (**B**) creatinine in the 5/6-nephrectomized and normal rats that had been injected with saline, ZnSO_4_, or rHuEPO on day 25, 32, and 39, respectively. The results are the mean ± SD for each time point (*n* = 6).

The blood urea nitrogen (Figure 5A) and creatinine (Figure 5B) of the 5/6-nephrectomized rats were elevated approximately 2.2-fold and 1.6-fold, respectively, compared with those of normal rats 25 days after surgery. The results indicated that 25 days after surgery, the 5/6-nephrectomized rats became uremic and continued until day 46. However, injection of ZnSO_4_ or rHuEPO had no effect on changing either the blood urea nitrogen or the creatinine level.

### 2.6. In Vitro Comparison of Zinc- and rHuEPO-Induced Erythropoiesis in Rat Bone Marrow Cells

**Figure 6:**
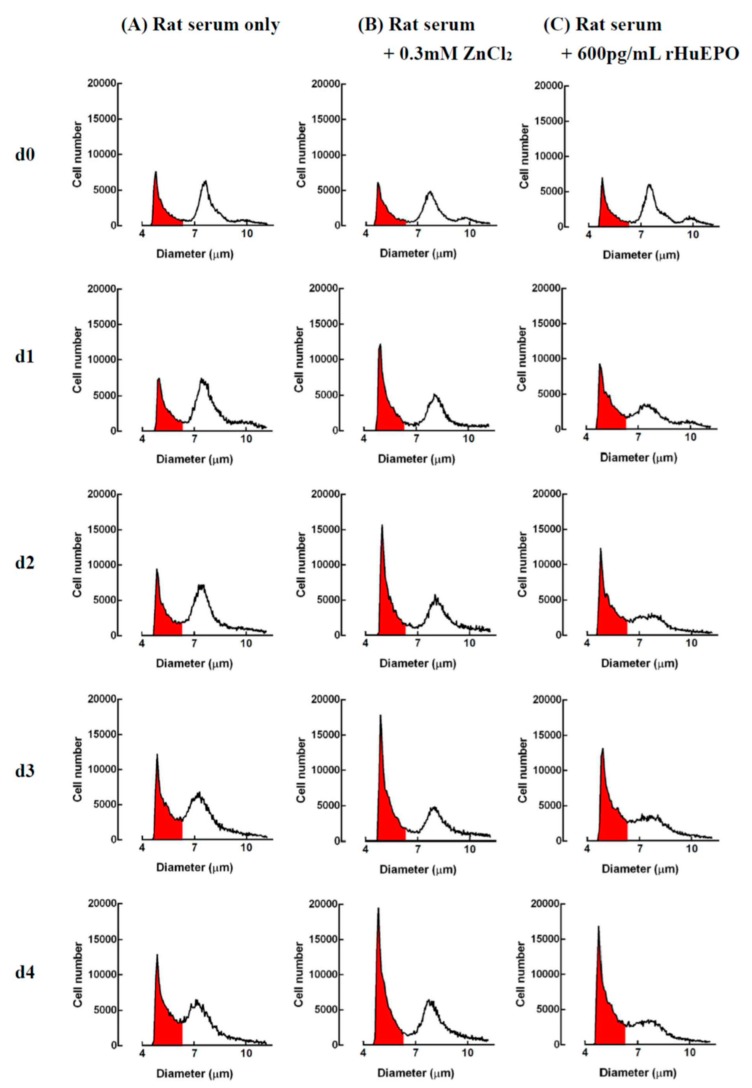
Representative profile of rat bone marrow cell suspensions cultured with (**A**) rat serum only, (**B**) 0.3 mM ZnCl_2_ + rat serum, and (**C**) 600 pg/mL rHuEPO + rat serum. After 1, 2, 3, and 4 days of culture, the cells were harvested, and the cell size and number were measured using an electron counter. At day 0, the rat bone marrow cells could be separated into three groups, the (a), (b), and (c) groups, which had peaks at 5.1, 7.4, and 8.5 μm, respectively. From day 1 to day 4, the number of (a) group (5.1 μm) cells significantly differed among the three different experimental groups. The differences in the 5.1-μm cells were calculated and are shown in Figure 7. No significant differences in the (b) and (c) group cells among the three different experimental groups were found. The data presented are from one representative experiment of three independent experiments.

**Figure 7:**
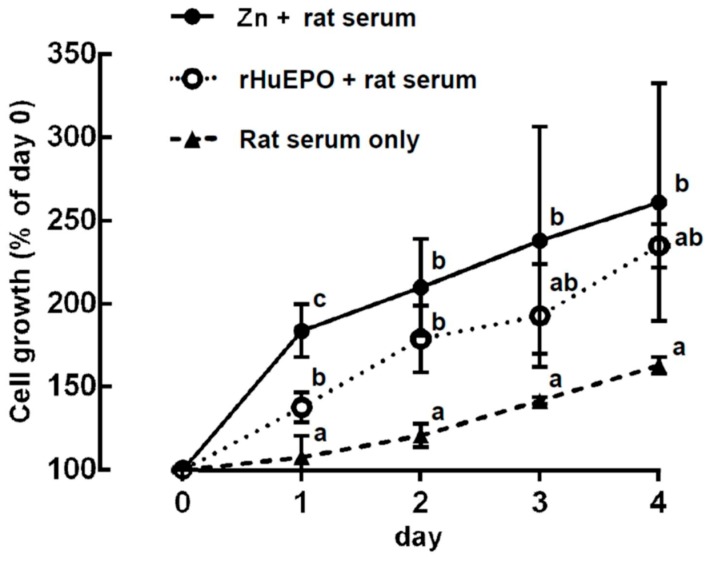
Comparison of cell growth induced using ZnCl_2_ or rHuEPO supplementation in rat bone marrow cells. Rat bone marrow cells were cultured in a suspension in medium with serum only, 0.3 mM ZnCl_2_ + serum, or 600 pg/mL rHuEPO + serum for 4 days. At each day, cell size and number of harvested cells were measured as shown in Figure 6. The cell growth is expressed as the ratio of the number of 5.1-μm cells at the start of the culture compared with that of the controls. a, b, c: Significant difference (*p* < 0.05, *n* = 6) among different experimental groups on the same day. The data presented are from one representative experiment of three independent experiments.

A representative profile of rat bone marrow cell suspensions cultured with serum only, zinc, or rHuEPO supplementation is shown in Figure 6. The rat bone marrow cells could be separated into three groups based on their cell sizes. After suspension culture with (a) serum only, (b) 0.3 mM ZnCl_2_ + serum, or (c) 600 pg/mL rHuEPO + serum, respectively, for 4 days, only the (a) group cells (median cell size, 5.1 μm) showed significant changes among the three different experimental groups. A previous study [15] indicates that the increase in 5.1-μm cells in the rat bone marrow cell suspension culture with ZnCl_2_ come from newly proliferated immature reticulocytes. In addition, the growth of immature reticulocytes in a rat bone marrow cell culture could be expressed by the increase in the 5.1-μm cells. Based on the change in the 5.1-μm cells shown in Figure 6, zinc- and rHuEPO-induced erythropoiesis are demonstrated in Figure 7. The figure indicates that after 1 day of culture, the cell growth in the ZnCl_2_-supplemented group was significantly higher than that in the rHuEPO- and saline-supplemented groups (184 ± 16%, compared with 138 ± 9% and 108 ± 13%, respectively). After 2 days of culture, both the ZnCl_2_- and rHuEPO-supplemented groups had significantly higher growth rates than the saline group (210 ± 29% and 179 ± 0%, compared with 121 ± 7%, respectively). After 3 and 4 days of culture, the ZnCl_2_-supplemented group still had a significantly higher cell growth rate than the saline group (262 ± 71% versus 142 ± 2%). However, there was no significant difference in cell growth between the ZnCl_2_- and rHuEPO-supplemented groups.

### 2.7. Effect of ZnCl_2_ Supplementation on the Growth of 5.1-μm Cells (Immature Reticulocytes) and Production of EPO from Rat Bone Marrow Cells

**Figure 8:**
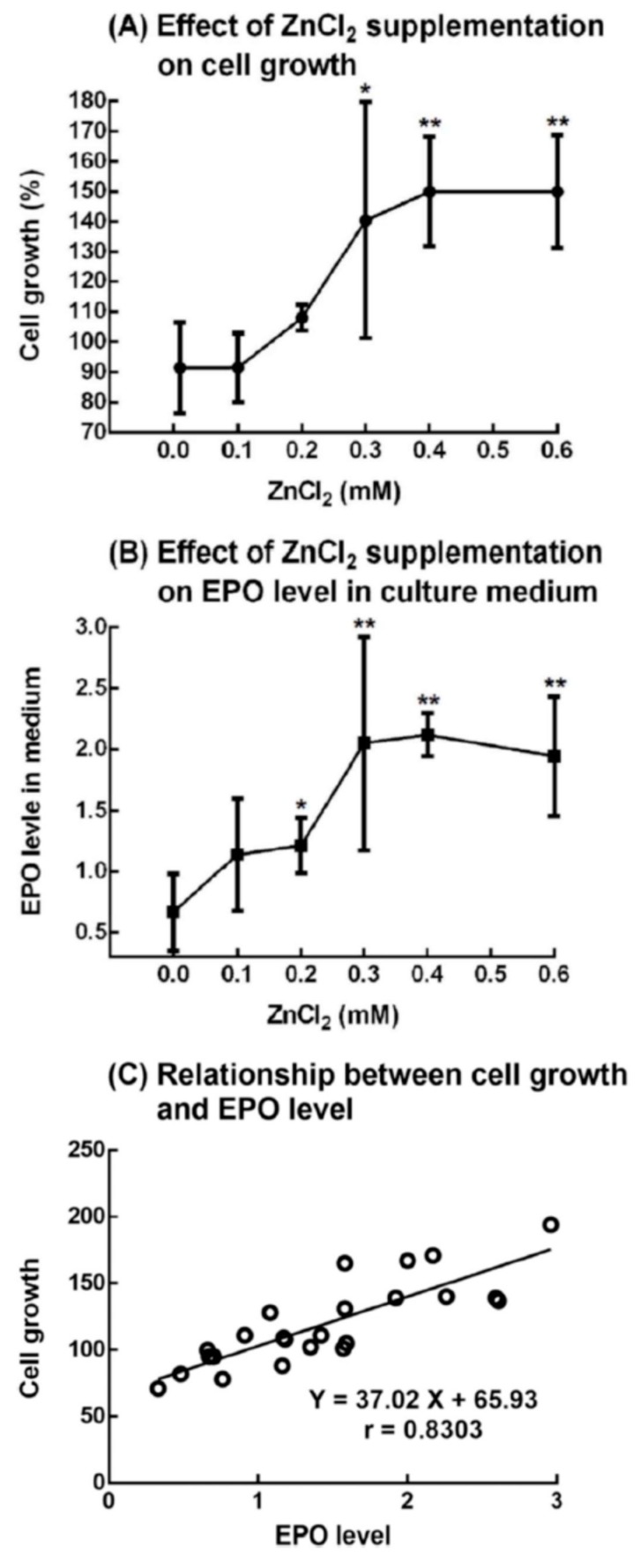
Effect of ZnCl_2_ supplementation on the growth of 5.1-μm cells (immature reticulocytes) and production of EPO from rat bone marrow cells. Rat bone marrow cells in the medium and rat serum were suspension-cultured with different concentrations of ZnCl_2_. The group without the supplementation of ZnCl_2_ (zinc concentration in the medium = 0.01 mM) was referred to as the control group, and the ZnCl_2_-supplemented groups (zinc concentrations of 0.1 to 0.6 mM) were referred to as experimental groups. After 1 day, the cultured cells were harvested: (**A**) Effect of ZnCl_2_ supplementation on cell growth. The cell growth is expressed as the ratio of the number of 5.1-μm cells at day 1 to that at day 0. (**B**) Effect of ZnCl_2_ supplementation on EPO levels in the culture medium. The EPO level is expressed as the ratio of EPO concentration at day 1 to that at day 0. (**C**) Relationship between cell growth and EPO level. The results are the mean ± SD from four independent experiments. * *p* < 0.05; ** *p* < 0.01: Significant difference between the ZnCl_2_ supplemented and control groups (*n* = 4).

Rat bone marrow cells in the medium and 10% rat serum were suspended in different concentrations of ZnCl_2_. After 1 day, significant cell growth was observed in the 0.3 to 0.6 mM ZnCl_2_-supplemented groups compared with the control group (zinc concentration = 0.01 mM) (Figure 8A). At day 0, the EPO concentration in the culture medium of all groups was found to be 443 ± 168 pg/mL (*n* = 28). We measured the EPO concentration in the rat serum and found that the EPO in the suspension culture came from the added rat serum. After 1 day of suspension culture, the EPO concentration in the culture medium in the control and experimental groups changed. The ratio of EPO concentration at day l to day 0 was calculated and expressed as the “EPO level in medium” shown in Figure 8B. In the 0.3 to 0.6 mM ZnCl_2_-supplemented groups, the EPO levels in the culture medium were significantly higher than those in the control group. It was also found that the cell growth was well correlated with the EPO level in the culture medium (Figure 8C).

## 3. Discussion

Anagnostou et al. [16] showed that the removal of 5/6 of the renal mass of rats in a two-stage procedure caused them to have elevated blood urea and mild to moderate anemia for at least 3 weeks. Kawamura et al. [20] indicated that 25 days after the operation, plasma urea nitrogen in 5/6-nephrectomized rats was increased approximately 2.5-fold, and the red blood cell count fell to 85% of that of the control. Our experiment showed similar results: the blood urea nitrogen and creatinine of the 5/6-nephrectomized rats were elevated to approximately 2.2-fold and 1.6-fold, respectively (Figure 5A,B); the RBC level dropped to 85 ± 4% (Figure 1A); and the anemic period continued for at least 3 weeks, from day 25 to day 46 (Figure 4A).

When these uremic and anemic 5/6-nephrectomized rats were injected with a ZnSO_4_ solution, both the single injection (Figure 1B) and three consecutive injections of zinc (Figure 4A) increased their RBC levels from approximately 85% to 95% of that of the sham-operated rats. In a previous paper [15], we showed that zinc stimulates RBC formation in PHZ-induced anemic rats. In the present paper, we further confirmed that zinc also stimulated RBC formation in 5/6-nephrectomized rats.

Kawamura et al. [20] showed that injection of rHuEPO into 5/6-nephrectomized rats induced a marked increase in the RBC count, hematocrit, and hemoglobin concentration. In the present paper, we compared the effect of ZnSO_4_ and rHuEPO injection on 5/6-nephrectomized rats. Both the in vivo result (Figure 4) and the in vitro experiments (Figure 7) indicated that zinc supplementation had the same effect in relieving anemia as supplementation with rHuEPO.

To know the effect of zinc supplementation on renal anemia, in the present report, 2.8 mg zinc/kg body weight was injected to the 5/6-nephrectomized rats. This was based on the results of our previous study [15], which showed that zinc stimulates RBC formation in PHZ-induced anemic rats in a dose-dependent manner, and that 2.8 mg zinc/kg body weight is an appropriate dose. It has been found that the intraperitoneal injection of median lethal dose (LD_50_) of zinc salts in rats is 28–73 mg/kg [15]. Therefore, the zinc level injected in the present study (2.8 mg/kg) was substantially lower than the reported LD_50_.

In a previous paper [15], rat bone marrow cells were cultured with rat serum and treated with different levels of zinc. After 1 day the harvested medium was subjected to a “Mouse” Erythropoietin Immunoassay kit (Quantikine^®^ELISA) to measure the EPO level. However, no EPO was detected in the culture medium. In the present study, we used a “Rat” EPO ELISA Kit (Elabscience^®^). Figure 8 shows the results of culturing rat bone marrow cells with rat serum and different concentrations of ZnCl_2_. This time, we were able to detect the EPO level in the culture medium in the control and experimental groups. As shown in Figure 8A, cell growth of the new RBCs significantly increased by approximately 1.5-fold after the rat bone marrow cells were supplemented with 0.3–0.6 mM ZnCl_2_. At the same time, the EPO level in the medium also significantly increased with 0.2–0.6 mM ZnCl_2_ (Figure 8B). Figure 8C shows that the cell growth was well correlated with the EPO level in the culture medium. In addition to this in vitro experiment, we also measured the EPO level in vivo in the plasma of the 5/6-nephrectomized rats that had been injected either with a ZnSO_4_ solution or saline (Figure 3). We found that the ZnSO_4_-injected 5/6-nephrectomized rats had a 1.7-fold higher EPO level than those injected with saline.

Based on the in vitro and in vivo experiments, we found that zinc stimulated the formation of EPO when administered as a supplement to rat bone marrow. Moreover, the level of EPO produced was dependent on the concentration of the zinc supplemented. In mammals, tissue-specific expression of the EPO gene is tightly controlled, and in adults, the kidneys are the main site of production of circulating EPO [22,23]. In addition to the kidney, EPO is also expressed in nonrenal sites, including the liver, brain, spleen, lung, and testis [24]. Both *EPO* mRNA and EPO protein are expressed in mouse and human erythroid progenitors [25,26]. Bone marrow macrophages were found to exhibit erythropoietin gene expression [27]. In our present experiment, “whole” bone marrow cells were used as material, and it is not clear now which cells in the bone marrow produced EPO. In a previous study [15], it was found that in vitro erythropoiesis using zinc in rat bone marrow cells was inhibited by the soluble EPO receptor or rat EPO antibody; in the present study, we further found that EPO was produced in a dose-dependent way when rat bone marrow cells were supplemented with zinc. Here, we present a hypothesis that there is a two-stage mechanism involved in zinc-induced erythropoiesis. In the first stage, zinc stimulates the rat bone marrow cells to produce EPO. In the second stage, EPO activates erythroid progenitors to reproduce erythrocytes. The mechanism of action of EPO to induce erythropoiesis is known [22,28]. However, how zinc induces rat bone marrow cells to produce EPO needs further study.

In conclusion, using 5/6-nephrectimized rats as a model for anemia related to CKD, we verified the effectiveness of zinc in correcting renal anemia. This finding suggests the possibility of treating patients with anemia through another pathway, i.e., producing EPO from bone marrow with a zinc supplement to stimulate RBC formation. The present study might be an important foundation for further studies of zinc supplementation as an inexpensive and reliable treatment for anemia

## 4. Materials and Methods

### 4.1. Animals

Normal, 5/6-nephrectomized, and sham-operated, 6-week-old male Sprague Dawley rats were purchased from BioLasco Taiwan Co., Ltd. (Taipei, Taiwan) (authorized distributor for Charles River Laboratories research models in Taiwan and Southeast Asia). The 5/6 nephrectomy was performed according to the Surgery Code: 56NEPHRES, using a two-stage procedure (standard method) by Charles River North American Research Models (Wilmington, MA, USA). After arriving in our laboratory, the rats were kept in the in-house animal facility and maintained at 22 ± 3 °C and 55 ± 5% relative humidity with a 12-h light–dark cycle. The rats were maintained under laboratory environmental conditions with ad libitum access to tap water and food. Animal use was reviewed and approved by the Animal Care and Use Committee at National Taiwan Ocean University, Keelung, Taiwan (No. 107029, 17 September 2018).

### 4.2. Effect of ZnSO_4_ Injection on the RBC Level of 5/6-Nephretomized Rats

Twenty-five days post surgery, the 5/6-nephrectomized rats were divided into two groups: the control group receiving saline solution and the experimental groups receiving 2.8 mg zinc/kg body weight of ZnSO_4_ solution intraperitoneally. After another 1, 2, and 4 days (at days 26, 27, and 29), the rats were sacrificed (*n* = 6, for each time point), and the RBC levels and other hematological metrics were measured using an automatic hematology analyzer (Excell 500; Danam Electronics, Dallas, TX, USA). Three independent experiments were performed.

### 4.3. Microscopic Observation of Rat Blood Cells Using New Methylene Blue Staining

Blood smears were prepared immediately after the blood sample collection, stained with new methylene blue [29], and subjected to microscopic observation. The images were observed using an inverted microscope (Olympus IX71, Olympus Optical Co, Ltd., Tokyo, Japan) and captured via an Olympus-DP70-Digital-microscope-Camera (Olympus Optical Co, Ltd., Tokyo, Japan). The images were then processed using DP Controller/Manager software (Olympus Optical Co, Ltd., Tokyo, Japan).

### 4.4. Comparison between the Injection of Saline, ZnSO_4_, or rHuEPO on the RBC Level of 5/6-Nephrectomized Rats

Twenty-five days post surgery, the normal and 5/6-nephrectomized rats were divided into three groups. Blood was drawn, and the control group was injected with sterilized saline. The experimental groups received 2.8 mg zinc/kg body weight of ZnSO_4_ solution and 100 IU/kg body weight of rHuEPO (Recormon (epoetin beta), Roche Diagnostics GmbH, Mannheim, Germany) via the intraperitoneal route (first injection). At day 32, rat blood was drawn for analysis, then saline, ZnSO_4_, or rHuEPO was injected (second injection). After 7 days (at day 39), the procedure was repeated (third injection). At day 46, the rats were sacrificed and blood was analyzed.

### 4.5. Determination of Blood Urea Nitrogen and Creatinine Contents

On days 0, 25, 32, 39, and 48 after surgery, the normal and 5/6-nephrectomized rats were anesthetized and blood was drawn using a cardiac puncture with heparinized syringes for the analysis of blood urea nitrogen and creatinine. Blood was collected into a microcentrifuge tubes and immediately mixed. Plasma was obtained by centrifuging 0.4 mL whole blood at 2000× *g* for 10 min and was used for the analysis of blood urea nitrogen and creatinine with a Hitachi 717 Chemistry Analyzer (Roche Diagnostic).

### 4.6. Comparison of Cell Growth Induced Using ZnCl_2_ or rHuEPO Supplementation in Rat Bone Marrow Cells

Aliquots of 0.15 mL of normal rat bone marrow cell suspension (60 × 10^6^ cells/mL) were added to equal volumes of DMEM/F12 medium supplemented with (a) 20% rat serum, (b) 20% rat serum + 0.6 mM ZnCl_2_, or (c) 20% rat serum + 1200 pg/mL rHuEPO. The cultures were incubated at 37 °C in an atmosphere of 5% CO_2_. At 0, 1, 2, 3, and 4 days, the cells from each well of each group were harvested. The number and size of the cells from the harvested cell suspension were determined with an electronic cell counter (Z2, Beckman Coulter, Hialeah, FL, USA). Cell growth was expressed as the ratio of the number of 5.1-μm cells relative to that of the control. Three independent experiments were performed.

### 4.7. Effect of ZnCl_2_ Supplementation on the Growth of 5.1-μm Cells (Immature Reticulocytes) and Production of EPO From Rat Bone Marrow Cells

Aliquots of 0.40 mL of normal rat bone marrow cell suspension (60 × 10^6^ cells/mL) were added to equal volumes of DMEM/F12 medium supplemented with 20% rat serum and with ZnCl_2_ (0, 0.2, 0.4, 0.6, 0.8, and 1.2 mM). After pipetting well, 0.40 mL of the cell suspension was removed and labelled the day 0 suspension. The remaining 0.40 mL cell suspension was seeded in a 24-well culture plate and incubated at 37 °C in an atmosphere of 5% CO_2_ for 1 day (labelled the day 1 suspension). The day 0 and day 1 suspension cells were subjected to measurements of cell number and size using an electronic cell counter. Part of the suspension cells were centrifuged at 2000× *g* for 10 min, and the supernatant (culture medium) was used for measuring EPO concentration using an ELISA kit. Four independent experiments were performed.

### 4.8. Determination of the EPO Concentration

EPO levels were determined spectrophotometrically (Multiskan GO Microplate Spectrophotometer, Thermo Scientific (Ratastie, Vantaa, Finland) using a Rat EPO ELISA Kit (Elabscience^®^, Houston, TX, USA) following the manufacturer’s procedure. Four groups of normal rats were used in this study, and the EPO concentration in the plasma was found to be 2353 ± 242 pg/mL (*n* = 6), 1380 ± 736 pg/mL (*n* = 11), 4430 ± 1680 pg/mL (*n* = 28), and 5587 ± 1359 pg/mL (*n* = 6). The normal rats had a mean EPO level between 1380 and 5587 pg/mL depending on the lot.

### 4.9. Statistical Analysis

The data are expressed as the mean ± standard deviation (SD). The significance of the experimental results was calculated using one-way analysis of variance, followed by the least significant difference post hoc test, using SPSS 22.0 (SPSS Inc., Chicago, IL, USA).

## Supplementary Materials

Supplementary materials can be found at https://www.mdpi.com/1422-0067/20/20/4985/s1. Supplemental Table S1. Hematology of the 5/6-nephrectomized rats injected with saline, ZnSO_4_, or rHuEPO; Supplemental Table S2. Hematology of normal rats injected with saline, ZnSO_4_, or rHuEPO.

## Abbreviations

CKD	chronic kidney disease
EPO	erythropoietin
rHuEPO	recombinant human erythropoietin
RBC	red blood cell
PHZ	phenylhydrazine
