# Supplementary material for: Effect of Zinc Supplementation on Renal Anemia in 5/6-Nephrectomized Rats and a Comparison with Treatment with Recombinant Human Erythropoietin
Source: Int J Mol Sci. 2019 Oct 9;20(20):4985. doi: 10.3390/ijms20204985 (PMC6829362; doi:10.3390/ijms20204985)
Supplement: Supplementary file 1 [file ijms-20-04985-s001.pdf]

Supplemental Table 1. Hematology of the 5/6-nephrectomized rats injected with saline, $ZnSO_4$, or rHuEPO.

	RBC (10^6 cells/mm^3)	HCT (%)	Hb (g/dl)	MCV (μm^3)	MCH (pg/cell)	MCHC (g/dl)
1. 5/6-nephrectomized rats (day 25)						
	5.58 ± 0.43	36.7 ± 2.5	15.2 ± 0.6	66 ± 4	27 ± 3	42 ± 3
2. After 1st injection (day 32)						
a. Saline	4.87 ± 0.20	33.7 ± 1.6	15.3 ± 0.6	69 ± 2	31 ± 1	45 ± 2
b. $ZnSO_4$	5.05 ± 0.59	33.6 ± 2.7	15.4 ± 0.8	67 ± 4	31 ± 2	46 ± 2
c. rHuEPO	5.28 ± 0.30*	35.1 ± 0.6	15.9 ± 0.6	67 ± 4	30 ± 2	45 ± 2
3. After 2nd injection (day 39)						
a. Saline	4.81 ± 0.20	30.9 ± 0.9	14.9 ± 0.3	64 ± 3	31 ± 1	48 ± 2
b. $ZnSO_4$	5.52 ± 0.17*	36.8 ± 0.7**	15.8 ± 0.1**	67 ± 2	29 ± 1	43 ± 1**
c. rHuEPO	5.36 ± 0.17	35.2 ± 0.2**	15.9 ± 0.3**	66 ± 2	30 ± 1	45 ± 1*
4. After 3rd injection (day 46)						
a. Saline	5.83 ± 0.18	37.0 ± 1.1	15.3 ± 0.3	64 ± 3	26 ± 1	41 ± 2
b. $ZnSO_4$	6.43 ± 0.37**	41.8 ± 2.7**	15.3 ± 0.6	65 ± 4	24 ± 1	37 ± 2**
c. rHuEPO	6.67 ± 0.39**	42.0 ± 1.8**	16.4 ± 0.5**	63 ± 3	25 ± 1	39 ± 1

1. Blood of the 5/6-nephrectomized rats (25 day post surgery) were drawn and for analysis. After the blood was drawn, the rats were divided into 3 groups, every 7 days injected with saline, $ZnSO_4$, or rHuEPO, respectively for 3 times.

2. RBC total red blood count, HCT hematocrit, Hb hemoglobin, MCV mean corpuscular volume (HCT/RBC × 10), MCH mean corpuscular hemoglobin (Hb/RBC × 10), MCHC mean corpuscular hemoglobin concentration (Hb/HCT × 100)

This table is representative of three different experiments.

* $P < 0.05$, ** $P < 0.01$, n=6, Significant differences between $ZnSO_4$- or rHuEPO- injected and saline-injected rats.

Supplemental Table 2. Hematology of normal rats injected with saline, $ZnSO_4$, or rHuEPO.

	RBC (10^6 cells/mm^3)	HCT (%)	Hb (g/dl)	MCV (μm^3)	MCH (pg/cell)	MCHC (g/dl)
1. Normal rats (day 25)						
	6.46 ±0.37	48.0 ±1.9	16.0 ±0.8	74 ±3	25 ±2	33 ±2
2. After 1st injection (day 32)						
a. Saline	5.63 ±0.52	37.7 ±1.9	15.1 ±0.8	67 ±2	27 ±1	40 ±2
b. $ZnSO_4$	5.75 ±0.39	40.3 ±3.1	15.6 ±0.5	70 ±3	27 ±2	39 ±3
c. rHuEPO	5.77 ±0.31	38.6 ±3.1	15.0 ±1.0	67 ±3	26 ±2	39 ±3
3. After 2nd injection (day 39)						
a. Saline	6.28 ±0.29	42.6 ±0.7	16.5 ±0.4	68 ±3	26 ±1	39 ±1
b. $ZnSO_4$	6.67 ±0.51	41.4 ±2.2	17.1 ±0.4	62 ±3*	26 ±2	41 ±2
c. rHuEPO	6.21 ±0.24	42.9 ±0.7	17.3 ±0.4	69 ±3	28 ±2	40 ±1
4. After 3rd injection (day 46)						
a. Saline	6.26 ±0.24	40.7 ±0.9	16.7 ±0.3	65 ±2	27 ±1	41 ±1
b. $ZnSO_4$	6.16 ±0.37	42.4 ±2.2	17.0 ±0.2	69 ±3*	28 ±1	40 ±2
c. rHuEPO	6.48 ±0.26	43.3 ±1.0**	17.6 ±0.5	67 ±1	27 ±1	41 ±1

Normal rats of same age and lot to those of the 5/6-nephrectomized were studied in a similar way as that in Table S1.
